# Effective Engagement Requires Trust and Being Trustworthy

**DOI:** 10.1097/MLR.0000000000000953

**Published:** 2018-09-13

**Authors:** Consuelo H. Wilkins

**Affiliations:** Department of Medicine, Vanderbilt University Medical Center and Meharry Medical College, Nashville, TN

Trust is essential to building and maintaining mutually respectful relationships, especially partnerships involving patients or community stakeholders and researchers, in which there is often an inherent imbalance of power. Patients and community members who are stakeholders in the design and conduct of health research rely on researchers’ honesty and willingness to protect them from harm. Although human research protections are in place for research participants, no such institutional protections are in place to provide oversight for patients and community partners involved in the research. Such vulnerability leads to lack of trust, which remains one of the most commonly cited barriers to public participation in research, especially among groups underrepresented in research.^1^ As public involvement in research continues to evolve, the types of relationships with researchers have changed from being participants in research projects to being consultants, advisory board members, and even patient and community principal investigators. These new roles and increasing power for stakeholders have not diminished the importance of trust. Instead, the need for trust is perhaps more important as patients and community members must navigate less familiar research settings and must depend on researchers to share resources, leadership, and decision-making.

The critical role of trust in public engagement is evident in publications emerging from newer approaches to engagement such as those used in the National Patient-Centered Clinical Research Network (PCORnet). The NYC Clinical Data Research Network modified its engagement strategies to facilitate involvement of people with limited trust and found lack of trust to be associated with concerns about data privacy and security, and lack of confidence that findings would be shared with the community.^2^ Within PCORnet, most networks identified trust as essential to achieving high levels of engagement and the need to build and nurture trust was clear.^3^ The recurring themes of trust and trustworthiness in public engagement also highlights the gap in our knowledge related to the underpinnings of trust in community-academic relationships, the need to measure, track, and improve trust, and the responsibility of researchers to become more trustworthy.

If building trust is widely recognized as essential to engagement, why after decades of community engagement in research, does trust remain so elusive? One challenge is its complexity. Trust is a multidimensional construct and though the term is used often, many people find it difficult to define. In general, trust refers to a firm belief in the reliability, truth, and ability or strength of someone or something.^4^ Trust has also been defined as the willingness to be vulnerable to the actions of another party, irrespective of the ability to monitor or control the other party.^5^ An individual may have trust in a specific researcher or abstract trust in the research enterprise. There are a number of factors that influence an individual’s level of trust in research including educational attainment, cultural beliefs, and personal as well as their community’s experiences with research. Despite its importance, little is known about strategies to improve trust among research participants and we are only beginning to consider trust among patients and community members who are involved in research roles as collaborators and partners.

The lack of validated tools to measure trust hampers our ability to determine the most effective ways to engender and improve trust. A systematic review identified 45 instruments that measure trust.^6^ The most frequently identified dimensions of trust in health systems are honesty, competency, fidelity, confidentiality, and global/system trust, whereas safety, fairness, and communication are more consistently identified dimensions of trust in the research setting. All but 2 of those 45 instruments were developed to measure trust in health systems or were designed for use by health professionals, not researchers. Because the relationships between health providers and patients are different from those between researchers and patient and community stakeholders, these existing instruments are not ideal for assessing trust in research partnerships. This difference was prominent in the work of the Greater Plains Collaborative, which contrasts trust in patient versus community engagement.^7^ Trust among patients was more likely built on interpersonal relationships, codified through formal processes, and unlikely to be transferred to others.

Interestingly, concerns about safety and fairness are also more common among racial and ethnic minorities^8^,^9^ and may reflect the underlying vulnerability that is inherent in research. Personal experiences with health systems, unequal access to health care, experiences with discrimination, and the history of unethical biomedical research likely contribute to the lack of trust among minorities.^1^,^10^,^11^ Other groups experiencing health inequities, such as individuals with lower educational attainment, also tend to be less trusting of research and the medical establishment. Consequently, the populations most likely to make research more relevant to them through engagement, are those less likely to engage, and lack of trust is a major reason why. Understanding this variability in levels of trust by population will require that trust measures be valid and relevant across populations. Engagement is required, then, even to develop effective trust measures.

Recognition of the different influencers and dimensions of trust is essential because trust instruments that measure competency, fidelity, and confidentiality may not capture lack of trust related to safety, communication, fairness, and negative intentions. In addition, dimensions of trust may present differently in community-academic partnerships, than among volunteers who are study participants. For example, a study participant who lacks trust related to fairness may be concerned that he is more likely to be randomized to placebo than a treatment deemed more beneficial, though a patient advocacy organization partnering in a research study may not trust the research team to fairly distribute resources.

Within the research setting, and perhaps more broadly in the health care system, the focus on trust is often on changing the patients, participants, or community members to make them more trusting. The attention is on the public’s lack of trust or distrust in research, and typically not on whether researchers are trustworthy. This framing, which may be subconscious, absolves researchers and the research enterprise of their roles in the relationship. The onus is on the public to change and be more trusting. Researchers and research institutions must place greater emphasis on being trustworthy and creating a culture that is inclusive and mutually respectful. This will require a shift in how researchers consider trust such that patient and community perspectives on trustworthiness of the research enterprise are more central.

**FIGURE 1:**
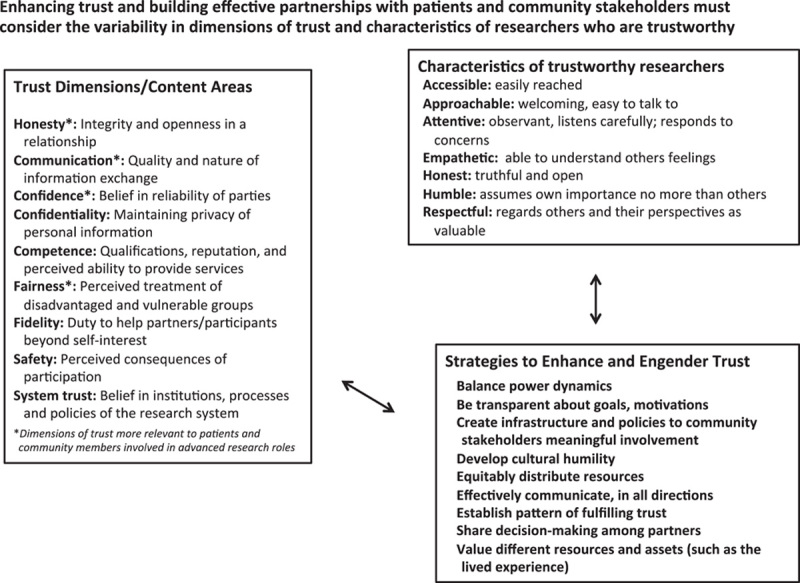
Conceptual framework for enhancing trust among and community-academic partnerships.

To enhance trust and build more effective patient and community-academic partnerships will require tools and strategies based on 3 concepts (Fig. 1):

1. The most important dimensions of trust differ based on the role in the research such that trust related to public involvement in more advanced research roles is often related to fairness and communication, and less related to competency and systems trust.
2. Characteristics of trustworthy researchers include being empathetic, accessible, approachable, honest, respectful, attentive, and humble. These characteristics are as important as, if not more than, technical competence and prestige of the research institution.
3. Strategies that enhance trust must build on the principles of community engagement^12^ including balancing power dynamics, equitable distribution of resources, effective bidirectional communication, shared decision-making, and valuing of different resources and assets (such as the lived experience and knowledge of group norms and perspectives).

Developing new tools to measure trust and testing interventions to improve trust must be done in partnership with patients and communities. This will ensure that instruments include content areas that reflect the research roles and include definitions and perceptions of trust relevant to underrepresented populations. Valid tools will improve understanding of trust and facilitate more precise assessment of strategies to amplify trust. Ideally new approaches to enhance trust simultaneously address researchers’ trustworthiness and create more opportunities for colearning.
